# Novel Anti-bacterial Activities of β-defensin 1 in Human Platelets: Suppression of Pathogen Growth and Signaling of Neutrophil Extracellular Trap Formation

**DOI:** 10.1371/journal.ppat.1002355

**Published:** 2011-11-10

**Authors:** Bjoern F. Kraemer, Robert A. Campbell, Hansjörg Schwertz, Mark J. Cody, Zechariah Franks, Neal D. Tolley, Walter H. A. Kahr, Stephan Lindemann, Peter Seizer, Christian C. Yost, Guy A. Zimmerman, Andrew S. Weyrich

**Affiliations:** 1 Molecular Medicine Program, University of Utah, Salt Lake City, Utah, United States of America; 2 Medizinische Klinik III, Universitaetsklinikum Tuebingen, Tuebingen, Germany; 3 Department of Surgery, University of Utah, Salt Lake City, Utah, United States of America; 4 Division of Haematology/Oncology, Program in Cell Biology, Department of Paediatrics, The Hospital for Sick Children, University of Toronto, Ontario, Canada; 5 The Petri-Hospital, Warburg, Germany; 6 Department of Pediatrics, University of Utah, Salt Lake City, Utah, United States of America; 7 Department of Internal Medicine, University of Utah, Salt Lake City, Utah, United States of America; National Institute of Allergy and Infectious Diseases, National Institutes of Health, United States of America

## Abstract

Human β-defensins (hBD) are antimicrobial peptides that curb microbial activity. Although hBD's are primarily expressed by epithelial cells, we show that human platelets express hBD-1 that has both predicted and novel antibacterial activities. We observed that activated platelets surround *Staphylococcus aureus* (*S. aureus*), forcing the pathogens into clusters that have a reduced growth rate compared to *S. aureus* alone. Given the microbicidal activity of β-defensins, we determined whether hBD family members were present in platelets and found mRNA and protein for hBD-1. We also established that hBD-1 protein resided in extragranular cytoplasmic compartments of platelets. Consistent with this localization pattern, agonists that elicit granular secretion by platelets did not readily induce hBD-1 release. Nevertheless, platelets released hBD-1 when they were stimulated by α-toxin, a *S. aureus* product that permeabilizes target cells. Platelet-derived hBD-1 significantly impaired the growth of clinical strains of *S. aureus*. hBD-1 also induced robust neutrophil extracellular trap (NET) formation by target polymorphonuclear leukocytes (PMNs), which is a novel antimicrobial function of β-defensins that was not previously identified. Taken together, these data demonstrate that hBD-1 is a previously-unrecognized component of platelets that displays classic antimicrobial activity and, in addition, signals PMNs to extrude DNA lattices that capture and kill bacteria.

## Author Summary

Platelets are small cells in the bloodstream whose primary function is to stop bleeding. In addition to their clotting functions, we show that human platelets stall bacterial growth. This inhibitory property of platelets is due to β-defensin 1, a small antimicrobial protein that kills bacteria. β-defensin 1 also induces white blood cells to discharge spider-like webs that trap and kill bacteria. Together, these findings indicate that human platelets use β-defensin 1 to fight off bacterial infection.

## Introduction

When bacteria enter the circulatory system, platelets are among the first cells they encounter[1]. It is well known that bacteria directly and indirectly induce platelet activation[2], but emerging evidence indicates that platelets also alter the activity of bacteria via the release of microbicidal proteins. Collectively, they are termed platelet microbicidal proteins (PMPs), and include chemokines, fibrinopeptides, and thymosin β-4[3]. Platelets deliver PMPs to sites of infection where they exert direct antimicrobial effects and potentiate the antibacterial properties of leukocytes[3], [4], [5], [6].

Whether PMPs represent the full repertoire of platelet antimicrobial peptides is not known. Other cells use defensins to counter invading bacteria[7]. Defensins are divided into α, β, θ family members that differ in structure, activity, and sites of expression[7]. Human α-defensins are very abundant in microbicidal granules of polymorphonuclear leukocytes (PMNs) and Paneth cells whereas β-defensins (hBD) are widely expressed in epithelium[7], [8], [9], [10], [11], [12]. This indicates that hBD's serve as a first line of innate defense against invasive pathogens[13]. Their primary mode of action is to insert into cell membranes, which allows hBD's to permeabilize and kill bacteria[14]. In general, hBD-1 is constitutively expressed while hBD's 2-4 are induced in response to infectious or inflammatory stimuli.

Other mechanisms of bacterial killing have recently been identified, including the formation of neutrophil extracellular traps (NETs) by PMNs[15]. NETs are lattices of DNA, histones and granule enzymes that are released when stimulated PMNs undergo a unique form of cell death[15], [16]. These DNA-rich NET complexes capture and kill bacteria. Platelets were reported to induce NET formation by PMNs in sepsis[17]. The signaling factors expressed by platelets that induce NET formation were not identified, however.

Here, we explored mechanisms by which platelets directly kill bacteria and, in parallel, actuate NET formation by PMNs. We found that human platelets store hBD-1 in extragranular compartments, identifying a previously unknown platelet antimicrobial factor. Platelets release hBD-1 when they are exposed to lytic toxins and hBD-1 retards the growth of clinical strains of *S. aureus.* In addition, we demonstrate for the first time that hBD-1 induces NET formation by human PMNs.

## Materials and Methods

### Ethics Statement

All studies were approved by the University of Utah Institutional Review Board committee. Written informed consent was provided by study participants and/or their legal guardians.

### Platelet and Bacterial Interaction Studies

Platelets were freshly-isolated from human subjects as previously described and exposed to CD45 positive selection, which effectively depletes contaminating leukocytes[18], [19]. The leukocyte-depleted platelet preparations were resuspended in M199 culture medium.

Unless otherwise indicated, *S. aureus* were isolated from clinical isolates of two sepsis patients and stored at −80°C. Twenty-four hours prior to each study, a portion of the bacteria were expanded on blood agar plates overnight at 37°C until they reached a stationary growth phase. The bacteria were resuspended in phosphate-buffered saline (PBS) and their concentration was determined by colorimetry (VITEK Colorimeter, bioMerieux, Inc., Durham N.C.). The *S. aureus* were then resuspended in M199 culture.

For each study, *S. aureus* (3×10^7^ total) was incubated in the presence or absence of freshly-isolated platelets (1×10^8^/ml) for four hours in M199 culture media unless otherwise indicated. After this incubation period, which provided an environment for exponential growth, the bacteria were serially diluted and 100 µl of each dilution was plated on blood agar plates. The bacteria were grown overnight at 37°C and the number of colony-forming units (CFU's) were counted the next morning.

In select studies, *S. aureus* was incubated in the presence of recombinant hBD-1 (PeproTech, Rocky Hill, NJ) or hBD-1 that was captured from platelet lysates. Recombinant hBD-1 was also pre-incubated with an anti-hBD-1 antibody (Abnova, Taipei City, Taiwan) or control IgG for 1 hour prior to being added to *S. aureus*. Based on preliminary studies testing the effectiveness of the anti-hBD-1 antibody in blocking hBD-1 induced NET formation, a final concentration of 20 µg/ml was chosen for all studies.

For the capture of hBD-1, immunoprecipitates were prepared from platelets (4×10^9^ total) that were lysed at 4°C in RIPA buffer (1 x PBS, 1% NP-40, 0.5% sodium deoxycholate, and 0.1% SDS). Insoluble cellular debri was cleared from the lysates by repeated centrifugation (13,000 x G, 10 minutes) at 4°C. Cleared lysates were incubated for 3 hours (4°C) with an antibody against hBD-1 (sc-20797, Santa Cruz Biotechnology, Santa Cruz, CA) or a rabbit IgG control (sc-2027, Santa Cruz). The antibodies were subsequently purified with protein A and G (A/G) coated agarose beads (4°C, overnight). The A/G beads were then centrifuged, pelleted, and washed with PBS. After 3 washes, immunoprecipitated proteins were eluted by adding 200 µl of glycine (100 mM, pH 2.5) to the beads for 10 minutes followed by centrifugation and isolation of the bead-free supernatant. The supernatant containing the eluted proteins was neutralized (pH 7.2) with TRIS and then incubated with *S. aureus* to measure growth as described above.

### mRNA Expression Analyses

Megakaryocytes or platelets were lysed in Trizol Reagent (Invitrogen, Carlsbad, CA) and RNA was extracted as previously described[18], [19]. Glycogen was added to the aqueous phase before precipitation with isopropanol to optimize RNA yields. The RNA was treated with DNAse (DNA free Kit, Ambion, Austin, Texas), precipitated with ethanol, and dissolved in 12 µl of RNAse-free water. Identical isolation methods were used to isolate RNA from HeLa cells, which served as a positive control for hBD family members.

RNA (1 µg) from HeLa cells or platelets was used to generate cDNA to characterize the expression of hBD-1, 2 and 3 using procedures similar to those previously published[18], [19]. Integrin αIIb was used as a positive control for megakaryocyte and platelet-specific RNA. The relative abundance of the defensin family members was measured by real-time PCR. The specificity of the amplicons for hBD-1, 2 and 3 was verified by agarose gel electrophoresis and subsequent sequencing of the products. Primer sets for these studies were as follows: hBD-1, forward-GTCGCCATGAGAACTTCCTACC, reverse- CTGCGTCATTTCTTCTGGTCAC; hBD-2, forward- GACTCAGCTCCTGGTGAAGCTC, reverse- ATGAGGGAGCCCTTTCTGAATC; hBD-3, forward- CAGCGTGGGGTGAAGCCTAGCA, reverse- TTTCTTCGGCAGCATTTTCGGC; and αIIb, forward- ACACTATTCTAGCAGGAGGGTTGG, reverse- CAGGGCTCAGTCTCTTTATTAGGC.

### Protein Expression Analyses

hBD protein expression was determined by immunocytochemistry and ELISA. The immunocytochemical studies were performed as previously described in platelets that were fixed immediately after they were isolated[20]. The intracellular pattern of hBD-1 expression was determined as previously described[20] using an antibody against hBD-1 (sc-20797; Santa Cruz) or its control (rabbit IgG sc-2027; Santa Cruz). Wheat germ agglutinin (WGA) (Alexa 555, Invitrogen), which stains granules and membranes of platelets[20], or phalloidin (Alexa 488, Invitrogen) was used as a counterstain.

To quantify hBD-1 protein levels, platelets were incubated with vehicle or a variety of agonists that included thrombin (Sigma-Aldrich, St. Louis, MO), thrombin receptor activating peptide (TRAP, Sigma), platelet activating factor (PAF; Avanti Polar Lipids, Alabaster, AL), *Escherichia coli-*derived lipopolysaccharide (LPS; Invivogen, San Diego, CA) or *S. aureus*-derived α-toxin (List Biological Laboratories, Campbell, CA). In select studies, platelets were preincubated with taxol (Molecular Probes, Eugene, OR) or nocodazole (Sigma) for 30 minutes prior to agonist stimulation (Figure S4). At the end of the incubation period, intact platelets were pelleted and lysed in RIPA buffer and the supernatants were collected. Cell lysates and supernatants were added in duplicate to commercially available ELISA plates specific for each of the defensin family members (Alpha Diagnostics International, San Antonio, TX). As previously described [18], [21], hBD-1 protein was also measured in platelet membranes and intracellular organelles that were isolated by centrifugation of lysates on sucrose gradients.

### Transmission Electron Microscopy

For the ultrastructural analyses, platelets and *S. aureus* were cultured in suspension followed by fixation in 2.5% glutaraldehyde in PBS buffer for at least 24 hours. The platelets and bacteria were then washed with 0.1 M phosphate buffer (pH 7.4) followed by dH2O by centrifugation at 800 x g (10 min). The samples were postfixed with 2% osmium tetroxide (60 min), washed twice with dH2O, dehydrated by a graded series of acetone concentrations (50%, 70%, 90%, 100%; 2×10 min each) and embedded in Epon. Thin sections were examined with a JEOL JEM-1011 electron microscope after uranyl acetate and lead citrate staining. Digital images were captured with a side-mounted Advantage HR CCD camera (Advanced Microscopy Techniques, Danvers, MA).

### NET Formation

The basic protocol for isolating PMNs from whole blood and imaging NETs was described in detail previously[16]. In brief, PMNs (2×10^6^/ml) were placed on glass coverslips coated with poly-L-lysine and incubated with recombinant hBD-1, hBD-2, or hBD-3 or its vehicle for one hour at 37°C. In some experiments, the recombinant defensins were pre-incubated with polymyxin B (10 µg/ml) for 15 minutes at room temperature before addition to PMNs. Recombinant hBD-1 was also pre-incubated with control immunoglobulin or a neutralizing anti-hBD-1 antibody for one hour before addition to PMNs. In addition, PMNs were incubated with platelet proteins released from hBD-1 or IgG immunoprecipitates as described above. LPS was used as a positive control for NET formation[16]. To examine the role of reactive oxygen species in hBD-1-dependent NET formation, PMNs were pretreated (30 minutes) with diphenylene iodonium (DPI, 20 µM) before addition of hBD-1. PMNs derived NETs were detected with a non-cell permeable DNA dye (Sytox Orange, Molecular Probes) while a cell permeable dye (Syto Green, Molecular Probes) was used to visualize nuclei[16].

### Quantification of Neutrophil Elastase Activity

PMNs (2×10^6^ cells /ml) in M199 were incubated at 37°C in 5% CO_2_/95% air with control buffer, LPS (100 ng/ml), or hBD-1 (100 ng/ml) for 60 minutes in a 24-well poly-L-lysine coated tissue culture plate. NET-associated neutrophil elastase activity was determined at selected time points as described[16].

### Statistics

Using a minimum of three studies, we calculated the mean ± standard error of the mean (SEM) for bacterial growth or hBD-1 protein levels for relevant figures. ANOVA's were conducted to identify differences among multiple experimental groups and if differences existed, a Student Newman-Keuls post-hoc procedure was used to determine the location of the difference. p<0.05 was considered statistically significant.

## Results

### Platelets Encapsulate S. *Aureus* and Inhibit Its Growth

**Figure 1:**
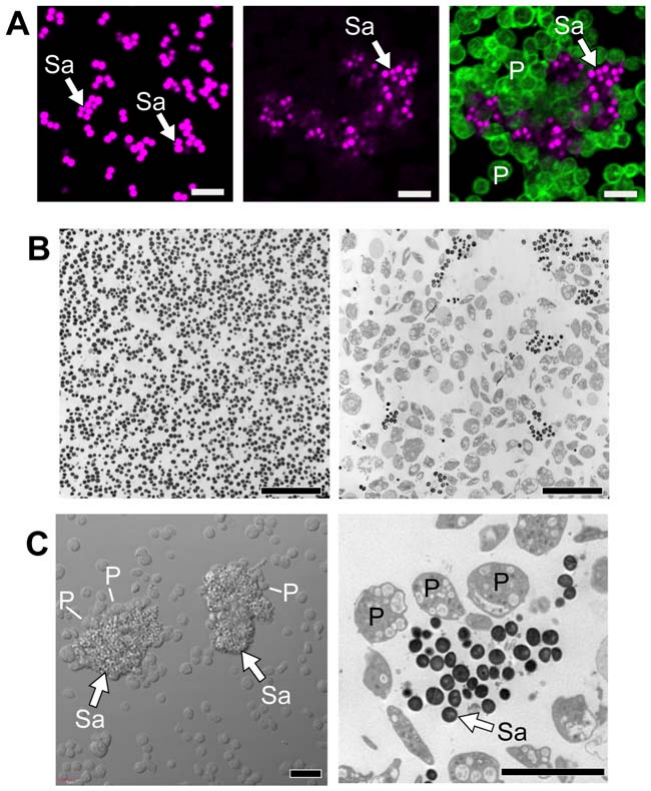
Platelets sequester *S. aureus*. Cultured *S. aureus* were incubated in the presence or absence of platelets (240 minutes). At the end of this period, an equal volume of fixative was added to the suspension and the samples were prepared for confocal (A) or transmission electron (B) microscopy. (A) The left panel shows *S. aureus* (magenta - stained with topro-3) only. The middle panel displays the pattern of *S. aureus* (magenta) growth in the presence of platelets, which are not visible in this panel but shown in an overlay that is displayed in the far right panel (bacteria, magenta; platelets, green – stained with phalloidin). Scale bars = 5 µm. (B) Transmission electron microscopy of *S. aureus* in the presence (right panel) or absence (left panel) of platelets. Scale bars = 10 µm. (C) Differential interference contrast (left panel) and transmission electron (right panel) microscopy of clusters of *S. aureus* (arrows) surrounded by platelets. Scale bars = 5 µm. The panels in figure 1 are representative of three to four independent experiments. Sa, *S. aureus*; P, platelets.

*S. aureus* expresses several proteins, such as clumping factor A and fibronectin-binding protein A, that mediate aggregation and activation of platelets [22], [23], [24]. Platelets also regulate the activity of *S. aureus*. In particular, there is evidence that platelets inhibit the growth of *S. aureus* [25], [26]. To examine the potential inhibitory role of platelets more closely, we incubated *S. aureus* with and without unactivated human platelets. In the absence of platelets, *S. aureus* grew uniformly in suspension culture over a four hour period (Figure 1A and 1B, left panels). In the presence of platelets, however, *S. aureus* were encapsulated in discrete clusters that were surrounded by platelets (Figure 1A–C). The majority of platelets in the culture were vacuolated and many of the platelets were visibly lysed.

**Figure 2:**
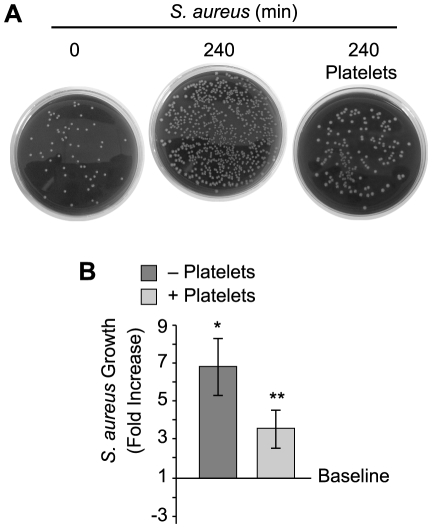
Platelets impede the growth of *S. aureus*. Cultured *S. aureus* were incubated in the presence or absence of platelets for 240 minutes and bacterial growth was then measured as described in Materials and Methods. (A) The blood agar plates are representative examples of four independent studies. (B) The bars in the lower panel represent the mean±SEM of the fold increase in *S. aureus* growth (240 minutes) in the presence or absence of platelets over baseline (horizontal line). The single asterisk indicates a significant increase (p<0.05) in growth over baseline. The double asterisks indicates a significant (p<0.05) reduction in *S. aureus* growth in the presence of platelets when compared to *S. aureus* growth by itself.

Although bacterial growth was noticeably impeded in the presence of platelets (Figure 1B), *S. aureus* ingestion by platelets was rare. Quantitative assessment demonstrated that platelets significantly inhibited the growth of two strains of *S. aureus* growth that were isolated from patients diagnosed with sepsis (Figure 2A–B and Figure S1). Platelets did not inhibit the growth of common laboratory strains of *S. aureus* (Figure S1). Likewise, the addition of thrombin to the cultures did not enhance the inhibitory effects of platelets on bacterial growth, which held steady for 8 hours but relinquished after 24 hours (data not shown).

### Platelets Express and Release β-defensin 1

**Figure 3:**
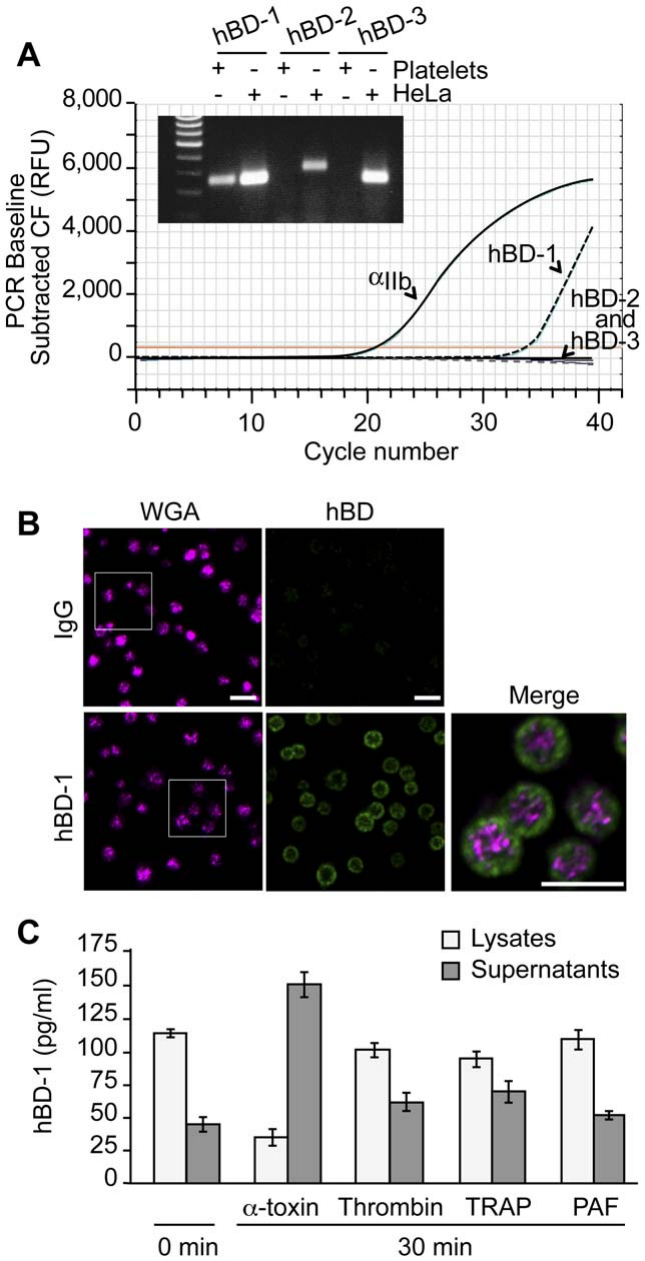
Platelets express and release β-defensin 1 in response to α-toxin. (A) The curves show real-time PCR results for integrin α_IIb_ and each of the defensin family members in platelets using the same template RNA. The inset shows semi-quantitative PCR for hBD-1, -2 and -3 in platelets and HeLa cells. This figure is representative of three independent experiments. (B) Immunocytochemical analysis of hDB-1 (green) and WGA in unstimulated platelets. Inset (top row, hBD-1): area selected for enlargement and merge. This figure is representative of five independent experiments. Scale bars = 5 µm. (C) hBD-1 protein in lysates and supernatants from platelets that were stimulated with α-toxin (50 ng/ml), thrombin (0.1 U/ml), TRAP (20 µM), or PAF (100 nM) for 30 minutes. The bars represent the mean±SEM of three independent experiments.

Platelets release a variety of anti-microbial mediators, however it is not known if defensins are part of this pool. Therefore, we screened for β-defensins in platelets and found mRNA for family member 1, but not 2 or 3 (Figure 3A). Precursor megakaryocytes also contained mRNA for β-defensin family 1 (Figure S2). Consistent with these findings, hBD-1 protein was readily detected in quiescent platelets (Figure 3B and 3C).

hBD-1 distributed to platelet compartments that were distinct from WGA staining of membrane regions (Figure 3B). We previously showed that WGA also co-localizes with the α-granule protein P-selectin[20]. Congruous with this staining profile, we were unable to detect hBD-1 in granules that were purified by subcellular fractionation (Figure S3). Furthermore, agonists that induce α-granule secretion (i.e., thrombin, TRAP, or PAF) did not appreciably elicit hBD-1 release from platelets (Figure 3C). We also found that microtubules, which modulate α-granule secretion[27], remained intact in platelets that were co-incubated with *S. aureus* (Figure S4A). Interruption of microtubular function with taxol or nocodazole in platelets had no effect on hBD-1 release in response to α-toxin, a pore-forming exotoxin that is derived from *S. aureus* (Figure S4B). Similarly, inhibition of microtubular function did not prevent platelets from limiting the growth of *S. aureus* (next section and Figure S4C).

### Platelet-derived β-defensin 1 Impedes the Growth of *S. Aureus*

**Figure 4:**
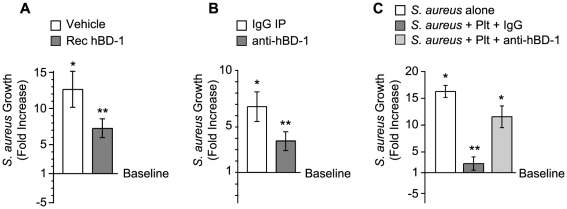
Platelet-derived β-defensin 1 inhibits the growth of *S. aureus*. (A) *S. aureus* growth was determined in the presence of recombinant hBD-1 (400 ng/ml) or its vehicle. The bars represent the mean±SEM of the fold increase in *S. aureus* growth (240 minutes) over baseline (horizontal line). The single asterisk indicates a significant increase (p<0.05) in growth over baseline. The double asterisks indicates a significant (p<0.05) reduction in *S. aureus* growth in the presence of recombinant hBD-1 when compared to *S. aureus* incubated with its vehicle. (B) *S. aureus* growth in the presence of immunoprecipitates that were captured from platelet lysates incubated with anti-hBD-1 or its control IgG. The bars represent the mean±SEM of the fold increase in *S. aureus* growth (240 minutes) over baseline (horizontal line). The single asterisk indicates a significant increase (p<0.05) in the growth of *S. aureus* that was treated with IgG immunoprecipitates over baseline. The double asterisks indicates a significant (p<0.05) reduction in *S. aureus* growth in the presence of anti-hBD-1 immunoprecipitates when compared to IgG immunoprecipitates. (C) The growth of *S. aureus* in the absence or presence of platelets that were co-incubated with a neutralizing antibody against hBD-1 or its IgG control. The bars represent the mean±SEM of the fold increase in *S. aureus* growth (240 minutes) over baseline (horizontal line). The single asterisk indicates a significant increase (p<0.05) in the growth of *S. aureus* alone or *S. aureus* co-incubated with platelets and anti-hBD-1 over baseline. The double asterisks indicates a significant (p<0.05) reduction in *S. aureus* growth in the presence of platelets incubated with control IgG when compared to *S. aureus* alone or *S. aureus* co-incubated with platelets and anti-hBD-1.

We determined if platelet-derived hBD-1 inhibits bacterial growth, an activity reported for recombinant hBD1. As expected, we found that recombinant hBD-1 retards *S. aureus* growth over a 4-hour period (Figure 4A). Similar inhibitory responses were observed when hBD-1 was captured from platelets by immunoprecipitation and then incubated with *S. aureus* (Figure 4B). In contrast, there was no inhibition by a control immunoglobulin immunoprecipitates (Figure 4B).

To investigate if endogenous hBD-1 protein inhibits bacterial growth, platelets were pre-incubated with a neutralizing anti-hBD-1 antibody or control immunoglobulin before the addition of bacteria. In these studies, platelets had a significantly reduced capacity to limit the growth of *S. aureus* in the presence of the hBD-1 neutralizing antibody (Figure 4C).

### β-defensin 1 Induces NET Formation by PMNs

**Figure 5:**
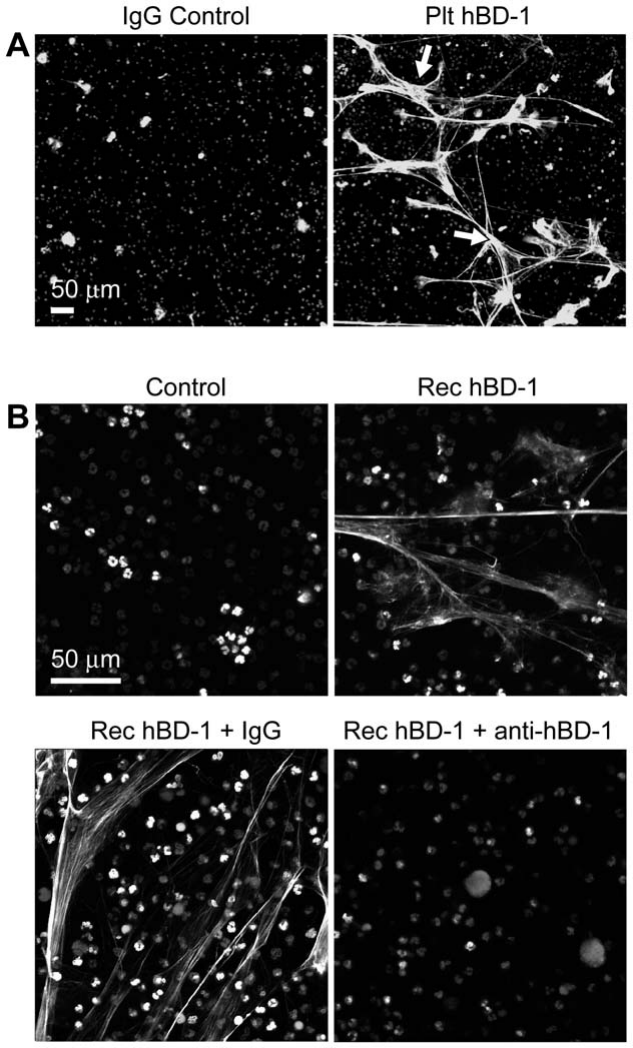
β-defensin 1 induces NET formation by PMNs. (A) NET formation in the presence of control immunoglobulin (left panel) or hBD-1 (right panel) immunoprecipitates that were captured from human platelets. The NETs (arrows) were detected by live cell imaging as previously described[16]. (B) NET formation in untreated PMNs (control) or PMNs incubated with 100 ng/ml of recombinant hBD-1 alone or in the presence of anti-hBD-1 or its control IgG. Figure 5A and 5B are representative of three independent experiments.

**Figure 6:**
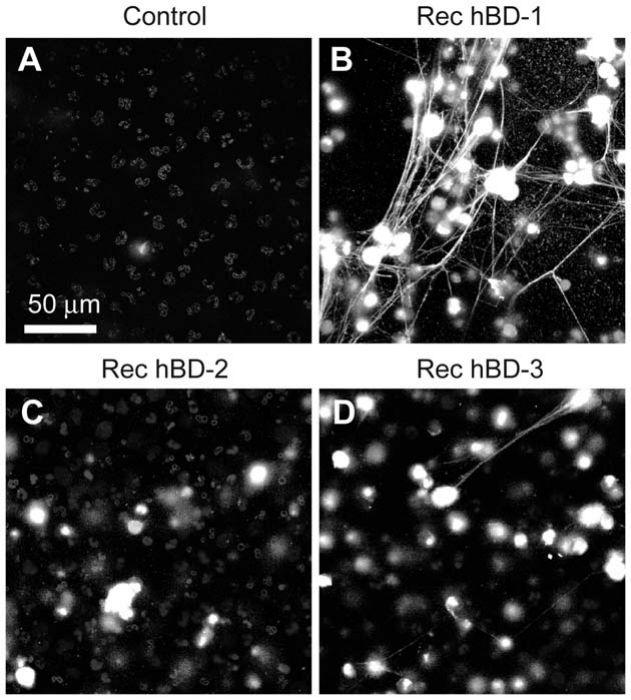
β-defensin 1, but not other β-defensin family members, induce PMNs to form NETs. PMNs were left untreated (control) or incubated with 100 ng/ml of recombinant hBD-1, hBD-2, or hBD-3. After 60 minutes, NETs (arrows) were detected by live cell imaging as previously described[16]. Increasing concentrations of hBD-2 or hBD-3 failed to induce NET formation (data not shown). Images are representative of three independent experiments.

We next asked if hBD-1 has novel antimicrobial activities and focused on the formation of NETs, a defensive function used by PMNs to trap and kill microbes in the extracellular milieu[16]. We first examined if platelet-derived hBD-1 could induce NET formation. hBD-1 harvested from platelet immunoprecipitates, but not control immunoprecipitates, induced robust NET formation (Figure 5A). Consistent with this finding, recombinant hBD-1 induced NET formation (Figure 5B). A neutralizing anti-hBD-1 antibody, but not control immunoglobulin, blocked hBD-1 dependent NET formation (Figure 5B) while having no effect on LPS-induced NET formation (data not shown). Unlike hBD-1, hBD-2 and hBD-3 did not induce appreciable NET production over a spectrum of concentrations (Figure 6 and data not shown).

**Figure 7:**
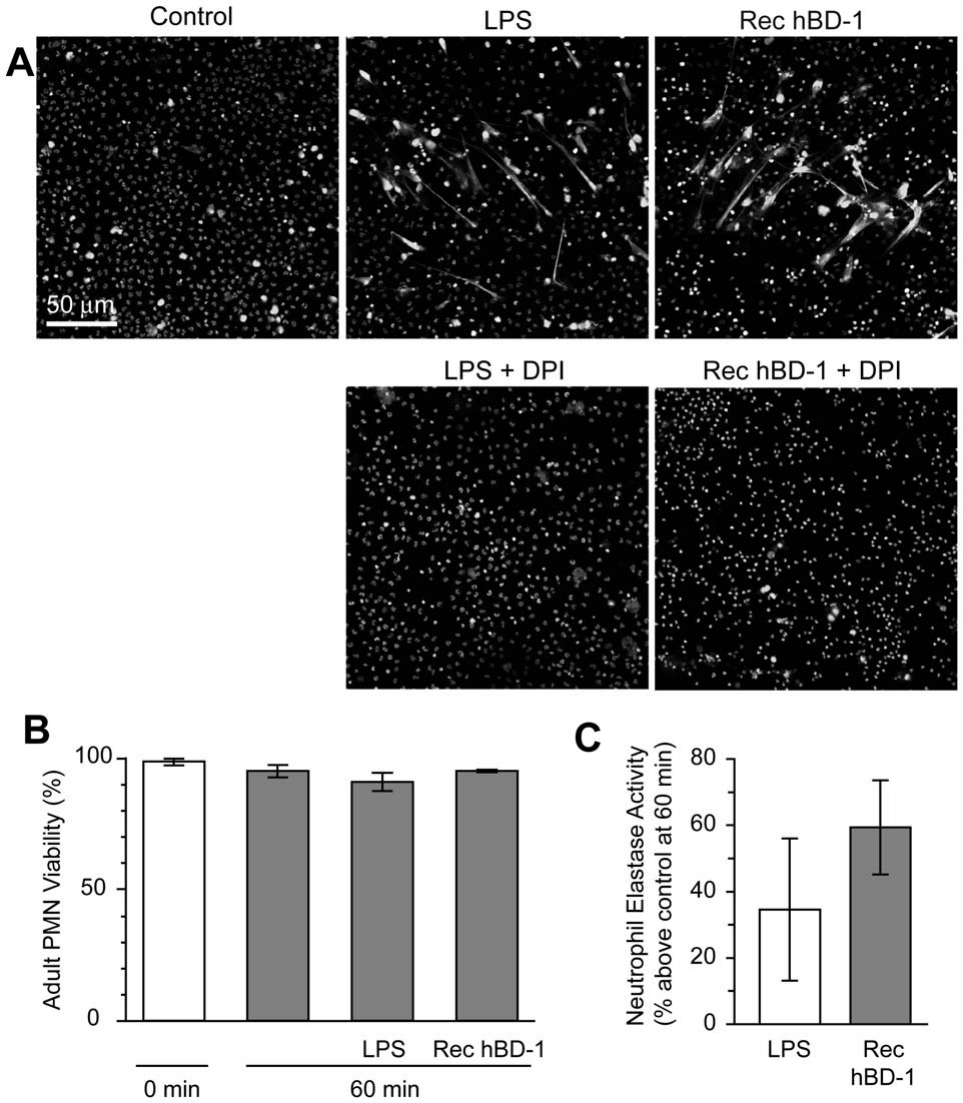
β-defensin 1 mediated NET formation is dependent on reactive oxygen species. (A) PMNs were incubated with diphenylene iodonium (DPI) and then left untreated (control) or stimulated with 100 ngl/ml of LPS or hBD-1 for 1 hour. Images are representative of three independent experiments. (B) PMNs were left alone or treated with 100 ng/ml of LPS or recombinant hBD-1 for 1 hour and then stained with trypan blue to determine cell viability. (C) PMNs were left alone or incubated with 100 ng/ml of LPS or recombinant hBD-1. After 1 hour, neutrophil elastase activity was measured. The bar graph represents the percent increase in neutrophil elastase activity over untreated neutrophils. The bars in panel B and C represent the mean±SEM of three independent experiments.

Inhibition of NADPH oxidase activity, a critical enzyme in the formation of reactive oxygen species (ROS), blocked LPS and hBD-1 induced NET formation compared to controls (Figure 7A). Polymyxin B, however, did not alter hBD-1 induced NET formation indicating that the effect of hBD-1 was not due to residual LPS contamination in the hBD-1 preparation (data not shown). Finally, hBD-1 significantly increased neutrophil elastase activity in the absence of appreciable cell death (Figure 7B and 7C).

## Discussion

In this report we show that platelets express hBD-1 that localizes to extragranular regions within the cell and is discharged into the supernatant in response to bacterial toxins. Platelet-derived hBD-1 directly inhibits the growth of strains of *S. aureus* isolated from patients with sepsis, suggesting that platelets may use hBD-1 to limit the growth of other bacteria as well. Moreover, hBD-1 induces PMNs to extrude NETs, identifying a new function of defensins in host defense. Together, these findings provide new insights into the antibacterial activity of platelets and establish a mechanism by which platelets may trigger NET formation. Although NET formation likely contributes to pathogen containment in humans, it is possible that under certain circumstances (i.e. clinical deterioration) it can lead to potentially deleterious pathologic platelet-leukocyte interactions in sepsis[17].

Mammalian defensins are small cationic peptides that have activity against a broad range of pathogens[7]. α-defensins are abundant in the microbicidal granules of PMNs and defensin alpha 1, also known as human neutrophil peptide-1, has been detected at the mRNA level by gene expression profiling in megakaryocytes[28]. β-defensins are generally present in skin and mucosal epithelia. β-defensins are phylogenetically older and new family members continue to be identified, with approximately 40 potential coding regions on the human genome[13]. hBD-1 was the first β-defensin discovered [29]. Although hBD-1 expression is primarily restricted to epithelium, it has been detected in peripheral blood [30] and was originally isolated from plasma filtrates of patients with end stage renal disease [29]. Here, we demonstrate that platelets express and release hBD-1 protein in response to *S. aureus*-derived toxins. Platelets have also recently been shown to express and release hBD-3 protein[31]. Interestingly, our data suggest that platelets may accumulate hBD-1 and hBD-3 protein through distinct mechanisms. In this regard, we detected mRNA for hBD-1 in precursor megakaryocytes and platelets suggesting that megakaryocytes transfer hBD-1 protein to platelets during thrombopoiesis. We did not, however, detect hBD-3 mRNA in platelets under the conditions of our experiments. This suggests that platelets may endocytose hBD-3 as they circulate in the bloodstream. Consistent with this possibility, Tohidnezhad and colleagues detected significant amounts of hBD-3 protein in plasma and platelets [31].

Unlike β-defensins 2–4, hBD-1 is constitutively produced by most epithelial cells[7], [8], [9]. It has also been detected in keratinocytes[32]. Thus, hBD-1 is positioned and ready to mediate innate host defense against pathogens in the gut, respiratory tract, oral cavities, and skin[7], [32], [33]. Megakaryocytes invest platelets with hBD-1 mRNA and hBD-1's basal protein expression in platelets indicates that it may also serve critical functions in defense against pathogens that gain access to the bloodstream. Platelets are immediate responders and the most abundant cell type to accumulate at sites of intravascular infection, which include infective endocarditis, suppurative thrombophlebitis, mycotic aneurysm, septic endocarditis, catheter and dialysis access site infections, and vascular prosthesis and stent infections[1], [2], [3]. Sequestration of bacteria and localized release of hBD-1 and other microbicidal proteins by platelets may limit the growth of pathogens at infected areas, providing time for leukocytes to gather and kill remaining bacteria.

*S. aureus* is one of the most common pathogens encountered by humans and a primary cause of infective endocarditis[2]. Our studies show that when platelets contact *S. aureus*, they encircle the pathogen and force it into encapsulated clusters. This may entrap and sequester the bacteria, reducing or preventing intravascular dissemination. The surrounding platelets are structurally altered, as evidenced by empty vacuoles. While hBD-1 is released under these conditions, our studies suggest that the mechanism is distinct from traditional secretory pathways in platelets since the defensin is basally localized in submembrane cytoplasmic domains rather than granules. In this regard, circumferential bands of microtubules are readily detected beneath the plasma membranes of platelets (Figure S3 and data not shown). This implies that *S. aureus* induces hBD-1 release by a mechanism that is distinct from granule secretion which is accompanied by microtubule reorganization, possibly by directly forming pores in platelet membranes. Indeed, some platelets were visibly lysed by *S. aureus,* which is perhaps a terminal event that discharges intracellular contents, including hBD-1, into the extracellular milieu. Furthermore, we found that α-toxin, which forms pores in cell membranes, evoked hBD-1 release by platelets in a microtubule-independent fashion while receptor-mediated agonists (i.e., thrombin, TRAP, or PAF) that typically induce α-granule secretion did not induce release of the defensin. Extragranular storage may guard against inappropriate release of hBD-1, which could have unwarranted cytotoxicity. Similar expression and release patterns are observed in epithelial cells, where hBD-1 is secreted independent of degranulation[33].

As part of the response to infection, host cells often internalize and kill bacteria. Thrombin-activated platelets are reported to engulf *S. aureus* [34], [35], but it is not clear that they form phagocytic killing chambers like PMNs and macrophages[36]. Internalization of *S. aureus* by platelets was rare under the conditions of our experiments (data not shown) and inhibition of platelet microtubule function, which facilitates *S. aureus* uptake in other cells[37], did not affect bacterial growth. This suggests that platelets limit *S. aureus* growth by quarantining the bacteria and locally releasing a variety of microbicidal proteins. Additional studies are required to determine if platelets display similar activities against *S. aureus* in more complex milieus that contain plasma and other cells. It will also be important to decipher why platelets limit the growth of clinical, but not laboratory, strains of *S. aureus*. In this regard, phenotypic and genotypic characterization of persistent *S. aureus* and determination of virulent signatures, which may or may not induce hBD-1 release from platelets, will be particularly informative.

To date, there are five distinct lineages of platelet microbicidal proteins (PMPs) that include: 1, platelet factor 4 and variants; 2, platelet basic protein and its proteolytic derivatives connective tissue activating peptide-3 and neutrophil activating peptide-2 (NAP-2); 3, regulated upon activation normal T cell expressed and secreted (RANTES); 4, thymosin β-4; and 5, fibrinopeptides [3], [6], [38]. In some cases, PMPs are cleaved into their active form by thrombin or other proteases in the immediate vicinity [3]. When platelets become activated they express surface P-selectin, which binds neutrophils, and release constitutively stored PMPs. Both platelet-neutrophil complexes and PMPs exert anti-microbial activity[6], [38], [39].

α-toxin induces the release of PMPs that possess staphylocidal activity [40]. Similarly, we demonstrate that platelets release hBD-1 in response to α-toxin indicating that hBD-1 is readily releasable from a cytoplasmic platelet compartment. PMPs and hBD-1 may have cooperative antibacterial activities. Like PMPs, we found that hBD-1 purified from platelets is capable of inhibiting the growth of *S. aureus*. PMPs permeabilize bacterial membranes in what appears to be a voltage-dependent manner[3] while defensins insert into bacterial membranes, inducing membrane depolarization and activation of lytic enzymes that permeabilize lipid bilayers[14]. Their precise modes of action at the bacterial cell wall, however, may not overlap completely. In side-by-side comparisons, Yeaman and colleagues [41] demonstrated that PMPs and neutrophil α-defensins disrupt *S. aureus* cytoplasmic membranes by distinct mechanisms. If the same holds true for β-defensins, PMPs and hBD-1 may attack bacteria from separate vantage points influencing the susceptibility of different bacterial strains to platelets. It is also possible that hBD-1 inhibits bacterial activity by cooperating with other factors that are yet to be identified. This notion is supported by the fact that neutralization of hBD-1 activity was only partially protective in preventing platelets from inhibiting bacterial growth.

It has been suggested that defensins have other functions besides direct microbial killing. Neutrophil-derived α-defensins modulate agonist-induced platelet aggregation and secretion[42]. β-defensins are chemotactic for monocytes, macrophages and dendritic cells[7], [43] and have been shown to differentially regulate the expression of numerous cytokines in human mononuclear cells[44]. Specifically, hBD-1 induces the expression of interleukin 8 and monocyte chemotactic protein-1[44]. It is well appreciated that platelets induce expression and other inflammatory gene products[45], [46]. Whether or not platelet-derived hBD-1 influences chemokine synthesis in target leukocytes is not known. Here, we show for the first time that hBD-1, but not other hBD family members, induces NET formation by PMNs, demonstrating an antimicrobial activity distinct from direct killing that amplifies the antibacterial properties of PMNs. NETs are web-like DNA structures that trap and kill bacteria. The DNA backbones of NETs are studded with histones, neutrophil elastase, myeloperoxidase, and bactericidal permeability increasing protein that together can degrade virulence factors and kill ensnared pathogens[15]. Although we demonstrate that platelet-derived hBD-1 induces NET formation, additional work is needed to dissect the exact roles of hBD-1 in eliciting NET formation by platelets that contact PMNs in human diseases such as sepsis. Previous studies have shown that LPS stimulated platelets induce PMNs to form NETs through mechanisms that remain unclear [17] [47]. Although hBD-1 may be involved in this process, LPS alone does not directly induce hBD-1 release by platelets (Figure S5). This suggests that multiple factors, or more potent lytics such as α-toxin, may be required to trigger hBD-1 release from platelets that subsequently stimulates NET formation by PMNs. Human BD-1 may also work in concert with *S. aureus*, which has recently been shown to directly induce NET formation by PMNs[48].

In summary, hBD-1 is basally expressed and released by platelets exposed to α-toxin. It is likely that hBD-1 cooperates with other PMPs to influence bacterial activity and growth, although their release patterns and potency against bacteria may be distinct from one another. By itself, hBD-1 directly inhibits the growth of gram-positive bacteria. Further, it has the novel capacity to engender PMNs to form NETs. The binary actions of hBD-1 are likely to play important roles in sepsis and other infectious diseases where platelets and neutrophils work together to trap, kill, and clear invading pathogens.

## Supporting Information

Figure S1 **Platelets impede the growth of clinical** ***S. aureus*** **strains.** Platelets were incubated with two laboratory strains of *S. aureus* or two strains of *S. aureus* (one non-methicillin resistant and one methicillin resistant) isolated from patients diagnosed with sepsis. The bars represent the mean±SEM (n = 3) of the fold increase in *S. aureus* growth (240 minutes) over baseline (horizontal line). The single asterisk indicates a significant increase (p<0.05) in growth over baseline. The double asterisks indicates a significant (p<0.05) reduction in *S. aureus* growth in the presence of platelets when compared to *S. aureus* growth by itself.
(EPS)

Figure S2 **Megakaryocytes express β-defensin 1** **mRNA.** The curves show a representative real-time PCR result for integrin αIIb and hBD-1 in megakaryocytes and platelets. The table below the figure identifies the Ct values for integrin αIIb and hBD-1 from two megakaryocyte cultures.
(TIF)

Figure S3 **β-defensin 1 is located in an extragranular compartment in platelets.** Platelets were exposed to nitrogen cavitation and their organelles (i.e., mitochondria and granules), membranes, and remaining cytosolic constituents were separated by sucrose gradients as previously described[49]. Each compartment was subsequently lysed in RIPA buffer and hBD-1 levels were determined. This graph is representative experiment of two independent studies.
(EPS)

Figure S4 **The antibacterial activities of β-defensin 1 are not dependent on microtubular reorganization.** (A) High resolution electron micrograph of a platelet that was co-incubated with *S. aureus*. The platelet ultrastructure is preserved with an intact microtubule coil (white arrow) and the absence of platelet projections, however, the platelet displays other features of activation that include increased vacuoles (black arrow). Scale bar = 1 µm. This micrograph is representative of numerous other platelets. (B) hBD-1 protein in platelet lysates and supernatants that were stimulated with α-toxin (50 ng/ml) in the presence of Taxol (50 µM), Nocodazole (Nocod; 10 µM), or their vehicle (control) for 30 minutes. The bars represent the mean±SEM of three independent experiments. (C) *S. aureus* was cultured for 240 minutes (gray bars) in the presence or absence of platelets that were pretreated with Taxol (50 µM) or Nocodazole (Nocod; 10 µM). The bars in the panel represent the mean±SEM (n = 3) of the fold increase in *S. aureus* growth (240 minutes) over baseline (horizontal line). The single asterisk indicates a significant increase (p<0.05) in growth over baseline. The double asterisks indicates a significant (p<0.05) reduction in *S. aureus* growth in the presence of platelets that were treated with or without Taxol or Nocodazole.
(TIF)

Figure S5 **LPS does not induce platelets to release β-defensin 1.** hBD-1 protein in lysates and supernatants from platelets that were stimulated with LPS (100 ng/ml) for 30 minutes. The bars represent the mean±SEM of three independent experiments.
(EPS)
