# Supplementary figures and images for: Novel Anti-bacterial Activities of β-defensin 1 in Human Platelets: Suppression of Pathogen Growth and Signaling of Neutrophil Extracellular Trap Formation
Source: PLoS Pathog. 2011 Nov 10;7(11):e1002355. doi: 10.1371/journal.ppat.1002355 (PMC3213094; doi:10.1371/journal.ppat.1002355)

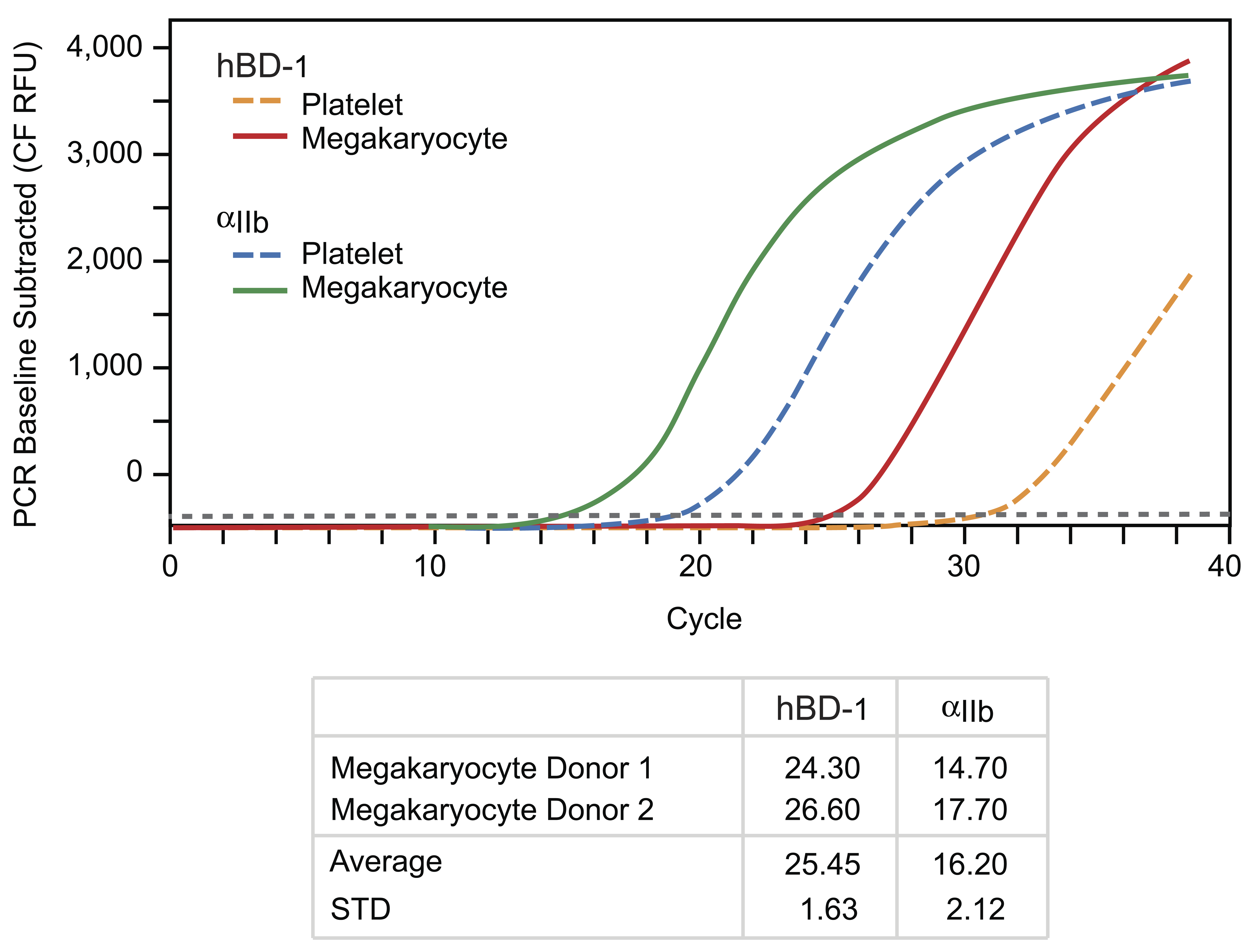

Supplement: Figure S2 — Megakaryocytes express β-defensin 1 mRNA. The curves show a representative real-time PCR result for integrin αIIb and hBD-1 in megakaryocytes and platelets. The table below the figure identifies the Ct values for integrin αIIb and hBD-1 from two megakaryocyte cultures. (TIF) [file ppat.1002355.s002.tif]

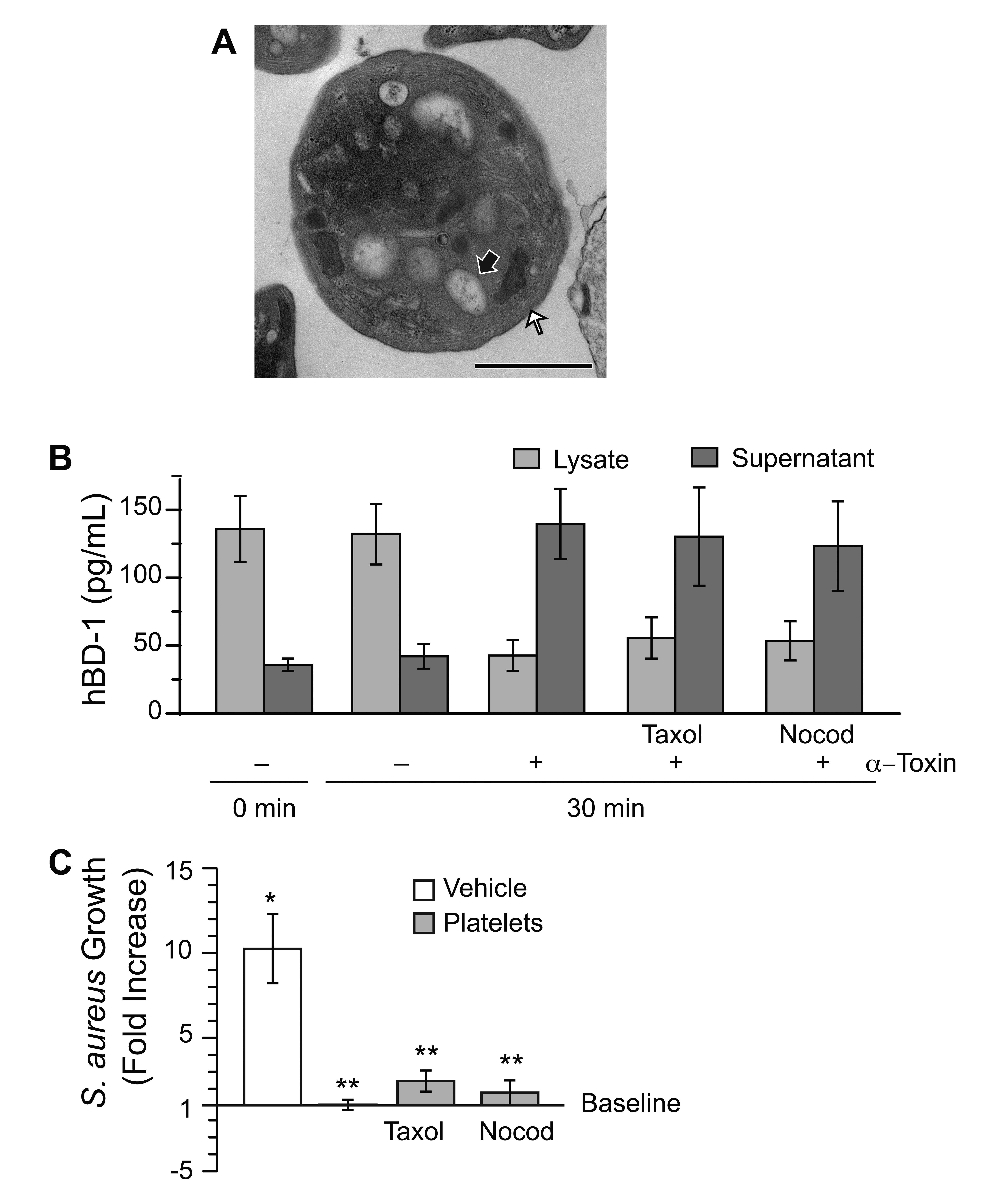

Supplement: Figure S4 — The antibacterial activities of β-defensin 1 are not dependent on microtubular reorganization. (A) High resolution electron micrograph of a platelet that was co-incubated with S. aureus. The platelet ultrastructure is preserved with an intact microtubule coil (white arrow) and the absence of platelet projections, however, the platelet displays other features of activation that include increased vacuoles (black arrow). Scale bar = 1 µm. This micrograph is representative of numerous other platelets. (B) hBD-1 protein in platelet lysates and supernatants that were stimulated with α-toxin (50 ng/ml) in the presence of Taxol (50 µM), Nocodazole (Nocod; 10 µM), or their vehicle (control) for 30 minutes. The bars represent the mean±SEM of three independent experiments. (C) S. aureus was cultured for 240 minutes (gray bars) in the presence or absence of platelets that were pretreated with Taxol (50 µM) or Nocodazole (Nocod; 10 µM). The bars in the panel represent the mean±SEM (n = 3) of the fold increase in S. aureus growth (240 minutes) over baseline (horizontal line). The single asterisk indicates a significant increase (p<0.05) in growth over baseline. The double asterisks indicates a significant (p<0.05) reduction in S. aureus growth in the presence of platelets that were treated with or without Taxol or Nocodazole. (TIF) [file ppat.1002355.s004.tif]
